# Astrocytes at the crossroads of obstructive sleep apnea and Alzheimer’s disease: from oxygen sensing to neurodegeneration

**DOI:** 10.1007/s11325-026-03651-w

**Published:** 2026-03-19

**Authors:** J. Cabot, J. B. Soriano, A. Alonso-Fernández, J. J. Rodríguez, J. J. Merino, L. Cànaves-Gómez, N. Gayà-Caro, X. Busquets

**Affiliations:** 1 Laboratory of Molecular Cell Biomedicine, Department of Biology, University of the Balearic Islands, Palma, Spain; 2 Faculty of Medicine, University of the Balearic Islands, Palma, Balearic Islands Spain; 3 Centro de Investigación Biomédica en Red de Enfermedades Respiratorias (CIBERES), Carlos III Health Institute (IS-CIII), Madrid, Spain; 4 Pulmonology Service, Son Espases University Hospital, Palma, Balearic Islands Spain; 5 Health Research Institute of the Balearic Islands (IdISBa), Palma, Balearic Islands Spain; 6 Functional Neuroanatomy Group, IKERBASQUE, Bilbao, 48009 Spain; 7 Department of Neurosciences, Medical Faculty, University of the Basque Country (UPV/EHU), 48940 Leioa, Spain; 8 Department of Pharmacology, Pharmacocognosy and Botanic, Complutense University of Madrid, Madrid, Spain; 9 Instituto Pluridisciplinar (UCM), Madrid, Spain; 10 Grupo de Medicina Regenerativa, Instituto de Investigación Sanitaria Hospital 12 de Octubre (imas12), 28041 Madrid, Spain

**Keywords:** Astrocytes, Sleep Apnea, Obstructive, Alzheimer disease, Hypoxia, Intermittent, Oxidative stress, Neurodegenerative diseases

## Abstract

**Purpose:**

Obstructive sleep apnea (OSA) is a highly prevalent sleep-related breathing disorder characterized by recurrent episodes of intermittent hypoxia and sleep fragmentation, and it has been increasingly linked to cognitive decline and Alzheimer’s disease (AD). Growing evidence suggests that vascular risk factors and sleep-related breathing disorders such as OSA may contribute to AD disease onset and progression. This review aims to explore astrocytes as a potential mechanistic link between OSA and AD.

**Methods:**

A literature search was conducted in the PubMed database using combinations of keywords including “astrocytes,” “obstructive sleep apnea,” “intermittent hypoxia,” “sleep-disordered breathing,” “Alzheimer’s disease,” and “dementia”.. Epidemiological, mechanistic, and clinical studies addressing the interplay between astrocyte function, sleep-disordered breathing, and neurodegeneration were reviewed and synthesized.

**Results:**

Astrocytes are increasingly recognized as oxygen-sensing cells capable of responding to fluctuations in oxygen availability. Under conditions of intermittent hypoxia, such as those occurring in OSA, astrocytic responses may become maladaptive, promoting oxidative stress and neuroinflammatory signalling. These processes overlap with key aspects of AD pathophysiology and may contribute to both disease initiation and progression. Although available evidence remains heterogeneous and not fully conclusive, epidemiological studies suggest a bidirectional association between OSA and AD.

**Conclusions:**

Glial maladaptation to intermittent hypoxia may represent a critical interface between sleep-disordered breathing and neurodegeneration. Targeting astrocyte-mediated mechanisms could offer new insights into the pathophysiological links between OSA and AD.

## Structured narrative review methodology

A literature search was conducted in the PubMed database from inception to March 2025 using combinations of keywords including “astrocytes,” “obstructive sleep apnea,” “intermittent hypoxia,” “sleep-disordered breathing,” “Alzheimer’s disease,”and “dementia” Boolean operators were applied to refine search strategies (e.g., “astrocytes AND Alzheimer’s disease,” “intermittent hypoxia AND Alzheimer’s disease, sleep-disordered breathing AND dementia).

**Fig. 1:**
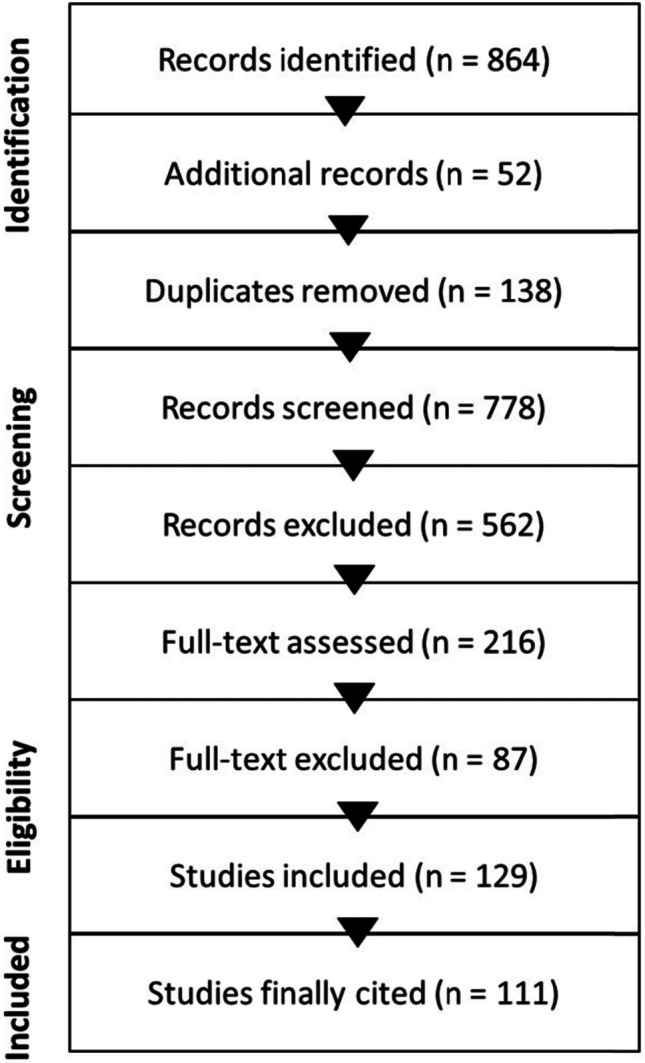
Study selection flow diagram. PubMed records were identified and screened using predefined search terms related to astrocytes, obstructive sleep apnea/intermittent hypoxia, dementia and Alzheimer’s disease. After duplicate removal and title/abstract screening, full texts were assessed for eligibility and the final set of studies was included in the qualitative synthesis

The initial search identified 864 suitable records. An additional 52 articles were retrieved through manual screening of reference lists. After removal of duplicates (*n* = 138), 778 records were screened by title and abstract. Of these, 562 were excluded for lack of relevance. A total of 216 full-text articles were assessed for eligibility, and 87 were excluded due to insufficient focus on astrocytes, absence of intermittent hypoxia/OSA relevance, lack of AD/dementia-related outcomes, or limited mechanistic or clinical contribution. Ultimately, 129 studies were included in the qualitative synthesis, of which 111 were cited in the final manuscript (Fig. 1).

## Alzheimer’s disease

Alzheimer’s disease (AD) represents the leading cause of dementia worldwide, with projections estimating up to 107 million affected individuals by 2050 [1]. However, the latest Global Burden of Disease official estimates available increase this projection considerably, up to 152.8 (130.8–175.9) million cases in 2050 [2].

The neuropathological hallmarks of AD include the progressive deposition of amyloid-β (Aβ) peptides and the formation of neurofibrillary tangles (NFTs) composed of hyperphosphorylated tau protein. These molecular alterations initiate a cascade of neurotoxic events [3–6], leading to cholinergic neuronal loss, synaptic dysfunction, and disturbances in neurotransmitter systems such as acetylcholine, GABA, glutamate, serotonin, and dopamine [7–10]. This cascade is known to be accelerated by certain genetic risk variants, with the apolipoprotein E (APOE) ε4 allele representing the most robust susceptibility factor [11]. In addition to these neuronal mechanisms, astrocytes have emerged as key players in AD pathophysiology, acting both as modulators of Aβ plaque dynamics and as sources of neuroinflammatory and oxidative responses [12, 13] contributing to cortical atrophy, neuroinflammation, and neuronal death [9, 14, 15].

## Obstructive sleep apnea

Obstructive sleep apnea (OSA) is a highly prevalent sleep-related breathing disorder characterized by recurrent upper airway collapse during sleep, resulting in intermittent hypoxia, hypercapnia, sleep fragmentation, and wide intrathoracic pressure swings. It has been estimated that 936 million (95% CI 903–970) adults aged 30–69 years (men and women) have OSA, and of them nearly half, that is 425 million (399–450), have moderate to severe OSA globally [16]. Beyond its clinical manifestations, OSA has cellular and molecular consequences. It is well established that each apnea–hypopnea event is associated with a transient decrease in oxygenation, reflected as a reduction in oxyhemoglobin saturation in polysomnographic recordings. Once the obstructive event resolves and ventilation resumes, oxygen saturation recovers, generating recurrent cycles of hypoxia-reoxygenation, referred to as intermittent hypoxia, which is characteristic of patients with OSA. This intermittent hypoxia is not an acute or isolated phenomenon, as it recurs nightly in the context of a chronic disorder. Therefore, OSA should be conceptualized as a condition of long-term intermittent hypoxia. In contrast, other respiratory diseases, such as chronic obstructive pulmonary disease (COPD), are more commonly associated with sustained hypoxia without repetitive hypoxia–reoxygenation cycles [17]. In this regard, cycles of hypoxia and re-oxygenation resemble ischemia–reperfusion injury and lead to excessive generation of reactive oxygen species (ROS), promoting oxidative stress [17–19].These events trigger the activation of inflammatory pathways (e.g., NF-κB, HIF-1α) and the release of cytokines such as IL-6 and TNF-α, which contribute to systemic inflammation and vascular dysfunction [20, 21]. At the neural level, OSA-induced intermittent hypoxia affects not only neurons but also astrocytes and microglia, leading to glial activation, impaired glutamate homeostasis, and disruption of neurovascular coupling [22–24].

## Astrocytes as oxygen sensors

Astrocytes are recognized as active sensors of the brain’s microenvironment, including oxygen fluctuations. Oxygen sensing by astrocytes is crucial because the brain is highly sensitive to hypoxia, and astrocytic responses help to maintain neuronal survival, regulate cerebral blood flow, and coordinate metabolic adaptation to low oxygen tension [25–27]. In particular, astrocytes act as oxygen sensors, translating oxygen fluctuations into metabolic and inflammatory responses, which under intermittent hypoxia become maladaptive, amplifying neuronal injury and potentially linking OSA to neurodegenerative processes [25, 28]. At the molecular level, astrocytes detect changes in oxygen availability through several mechanisms. One is through mitochondrial function increasing ROS production. These ROS act as signaling molecules that trigger downstream responses in astrocytes, including the activation of redox-sensitive transcription factors such as hypoxia-inducible factor 1α (HIF-1α). Stabilization of HIF-1α under hypoxic conditions promotes the transcription of genes involved in glycolysis, angiogenesis, and antioxidant defense, thereby adapting brain tissue to oxygen scarcity [29–32]. Oxygen-sensitive ion channels have been recognized as additional intermediate mechanisms. Astrocytes express potassium (K⁺) and calcium (Ca^2^⁺) channels whose activity is modulated by oxygen tension. In particular, hypoxia can inhibit potassium channels, leading to depolarization of the astrocytic membrane and subsequent calcium influx [25, 26, 33, 34]. Elevated intracellular calcium in astrocytes has been linked to the release of gliotransmitters such as adenosine triphosphate (ATP), glutamate, or lactate, which in turn modulate neuronal excitability and vascular tone [35, 36]. This suggests that astrocytes act as intermediaries in neurovascular coupling, allowing them to modulate vascular responses to hypoxia. For instance, hypoxic astrocytes can release vasoactive factors such as prostaglandins, nitric oxide, and ATP, leading to vasodilation and improved oxygen delivery to hypoxic regions [37–39].

## Astrocytes, AD and OSA

The involvement of astrocytes in AD has been recognized since Alois Alzheimer’s first descriptions of altered glial morphology associated with neuritic plaques and neuronal damage [40]. Subsequent research has consolidated the concept of astrocytes as active participants in AD pathophysiology [41–47]. Reactive astrogliosis is consistently reported both in patient samples and in animal models of AD [48–50]. However, recent evidence from human studies has revealed marked astrocytic atrophy in the entorhinal cortex of both Alzheimer’s disease (sporadic and familial) as well as in frontotemporal dementia, underscoring that astrocyte degeneration may represent a critical pathological feature in neurodegeneration [45–47]. Astrocytes appear to play a dual role: on the one hand, they contribute to limiting Aβ plaque burden, while on the other, their chronic atrophy (seen in some regions, while in the hippocampus a subpopulation shows astrogliosis) may exacerbate neuronal dysfunction. Experimental studies have shown that extracellular Aβ can trigger astrogliosis in vitro [51–53] and astrocytes are often located at the dense core and periphery of amyloid plaques, with their processes infiltrating the plaque structure [48, 52]. Furthermore, exposure of astrocytes to Aβ induces calcium oscillations, which enhance astroglial activation and foster neurotoxic signalling [53, 54].

In pathological contexts, astrocytic oxygen sensing may become maladaptive. One of the main deleterious consequences of OSA is intermittent hypoxia as a result of recurrent cycles of oxygen desaturation and reoxygenation. Astrocytes exposed to this environment show enhanced ROS production, sustained activation of HIF-1α, and exaggerated release of pro-inflammatory mediators [17, 20]. These alterations exacerbate oxidative stress and dysfunctional glucose metabolism, processes known to contribute to AD [55]. In AD models, dysregulated astrocytic responses to oxygen fluctuations have been linked to impaired clearance of amyloid-β, increased tau pathology, and compromised synaptic support [12, 13]. Repetitive cycles of intermittent hypoxia and re-oxygenation in OSA show a similar pattern found in ischemia–reperfusion injury. Astrocytic mitochondria, which normally fine-tune oxygen sensing through electron transport chain activity, become chronically dysregulated under these conditions, generating excessive ROS that damage astrocytic DNA, proteins, and lipids [53, 56]. A critical pathway in astrocytic oxygen sensing and redox signaling involves nicotinamide adenine dinucleotide phosphate (NADPH) oxidases (NOX enzymes). Unlike mitochondria, which generate ROS as a by-product of oxidative phosphorylation, NOX enzymes are dedicated ROS-producing systems. Astrocytes express several NOX isoforms, with NOX2 and NOX4 being the most prominent in the central nervous system [57, 58]. NOX2, typically localized at the plasma membrane, is strongly upregulated in reactive astrocytes in response to Aβ and pro-inflammatory cytokines. Activation of NOX2 increases superoxide and hydrogen peroxide production, which promotes astrocytic release of pro-inflammatory mediators and exacerbates synaptic dysfunction [53, 59, 60]. NOX4, mainly found in mitochondria and the endoplasmic reticulum, produces ROS constitutively and is particularly sensitive to hypoxic conditions. In AD models, NOX4 upregulation in astrocytes has been associated with mitochondrial dysfunction, altered calcium signalling, and enhanced tau pathology [59, 61].

In this regard, serum levels of NOX4 have been found to be increased compared with healthy control subjects and associated with the severity of OSA [62]. In this context, studies in a murine model of OSA conclude that brain NADPH oxidase serves as a critical source of superoxide in oxidative injury and hypersomnolence [63, 64]. A-930G polymorphisms of the p22phox gene may also play an important role in genetic susceptibility to OSA and may be involved in the development of oxidative stress in OSA patients [65].

The chronic astrocytic response is marked by reactive astrogliosis, characterized by hypertrophy and secretion of pro-inflammatory mediators (IL-1β, TNF-α, IL-6). These cytokines contribute to blood–brain barrier (BBB) dysfunction [66], and BBB dysfunction has been hypothesized to be a triggering factor in neurodegeneration. Recently, alterations in cerebrospinal fluid (CSF) Aβ42 and BBB dysfunction have been reported in patients with OSA [67]. Aquaporin-4 (AQP4) is the predominant water channel in the CNS, predominantly concentrated in the endfeet and fine processes of astrocytes. Evidence shows that AQP4 is essential for multiple astrocytic functions, including the regulation of cerebral water homeostasis, spatial buffering of extracellular potassium, modulation of calcium signaling, control of neurotransmission, synaptic plasticity, and the support of adult neurogenesis [68]. Under neuropathological conditions, AQP4 becomes involved in reactive astrogliosis and the release of pro-inflammatory cytokines, contributing to the onset and progression of several neurodegenerative and neuropsychiatric diseases such as AD, Parkinson’s disease, depression, and substance addiction [68].

In the context of AD, the regulation of AQP4 channel activity is one of the main mechanisms through which the glymphatic system eliminates Aβ and tau aggregates [13, 69, 70]. Since recent evidence from human studies has revealed marked astrocytic atrophy in the entorhinal cortex of AD patients [47], AQP4 channel activity could be inhibited and therefore compromise glymphatic clearance. In this regard, sleep fragmentation, as occurs in OSA, affects the glymphatic system through altered AQP4 expression in wild-type and 5xFAD mouse models [71]. Thus, astrocytes function as dynamic oxygen sensors through mitochondrial, ionic, and signalling pathways. Their ability to translate oxygen availability into metabolic, vascular, and inflammatory responses positions them as key regulators of brain resilience to hypoxia. When chronically challenged, as in OSA or AD, astrocytic oxygen sensing may shift from protective to pathogenic, underscoring the importance of targeting these pathways for therapeutic purposes.

Altogether, these mechanisms highlight the multifactorial interplay between intermittent hypoxia, astrocytic dysfunction, and neurodegeneration, as summarized in Table 1.

**Table 1:** Summary of mechanistic and clinical links between Alzheimer’s disease (AD) and obstructive sleep apnea (OSA)

Aspect	Description/Mechanisms	Key References
Pathophysiological connection	OSA and AD share systemic and cerebral alterations including intermittent hypoxia, oxidative stress, vascular dysfunction, and inflammation, leading to neuronal injury and cognitive decline	Lavie 2015; Ryan et al. 2005; Butterfield & Halliwell 2019
Astrocytes as oxygen sensors	Astrocytes sense oxygen changes through mitochondrial ROS, HIF-1α activation, and ion channel modulation, leading to gliotransmitter release and regulation of neurovascular coupling	Gourine & Funk 1985; Angelova & Abramov 2016; López-Barneo et al. 2016
Astrocytic maladaptation in OSA	Chronic intermittent hypoxia induces sustained ROS, HIF-1α activation, and pro-inflammatory cytokines (IL-1β, TNF-α, IL-6), promoting BBB disruption and synaptic dysfunction	Ryan et al. 2005; Lavie 2015; Jelic et al. 2010
NADPH oxidase (NOX) pathways	Astrocytic NOX2/NOX4 are major ROS sources under hypoxia and in AD; their up-regulation is linked to oxidative stress, mitochondrial dysfunction, and tau pathology	Sorce & Krause 2009; Nayernia et al. 2014; Ekin et al. 2021
Aquaporin-4 and glymphatic dysfunction	AQP4 mislocalization/altered expression in OSA or AD impairs glymphatic clearance of Aβ and tau, contributing to their accumulation; sleep fragmentation further disrupts AQP4-dependent fluid exchange	Kress et al. 2014; Nedergaard & Goldman 2020; Vasciaveo et al. 2023
Astrocytes in AD pathology	Astrocytes surround amyloid plaques and become reactive, showing Ca^2^⁺ oscillations and enhanced ROS signaling that aggravate neuronal damage	Nagele et al. 2004; Abramov et al. 2004; Verkhratsky et al. 2016
Shared oxidative stress	Intermittent hypoxia in OSA and Aβ accumulation in AD both elevate ROS, driving mitochondrial dysfunction, lipid peroxidation, and reduced antioxidant defenses	Zhang et al. 2012; Barceló et al. 2006; Poljsak & Milisav 2012
Epidemiological link	Meta-analyses and large population studies show OSA increases risk of dementia and AD (HR ≈ 1.3–1.5), with possible bidirectional association; prevalence of OSA is up to fivefold higher in AD patients	Yaffe et al. 2011; Emamian et al. 2016; Guay-Gagnon et al. 2022; Gribsholt et al. 2025; Wang et al. 2025
Sex differences	Women with OSA show a higher dementia incidence by age 80 than men, indicating greater susceptibility	Braley et al. 2024
Experimental evidence	Chronic intermittent hypoxia in AD mouse models enhances Aβ₄₂ accumulation, tau phosphorylation, and oxidative damage in hippocampus and cortex	Xu et al. 2004; Shiota et al. 2013; Kazim et al. 2021
Therapeutic implications	CPAP enhances oxygenation and mitigates oxidative stress, showing potential to lower AD risk and improve brain metabolism, while antioxidant therapies yield inconsistent results	Schulz et al. 2000; Alonso-Fernández et al. 2008; Dunietz et al. 2021; Fernandes et al. 2022;

## OSA and AD. Natural history and clinical evidences

A pioneering work by Ancoli-Israel et al. in 1991 concluded that there is a strong relationship between dementia and OSA when both conditions coexist [72]. Vascular risk factors are highly prevalent in OSA, and their impact is exacerbated by the pathophysiology of sleep-disordered breathing. Intermittent hypoxia and sleep fragmentation in OSA drive sympathetic overactivity, endothelial dysfunction, oxidative stress, and systemic inflammation, thereby worsening blood pressure control, insulin [73], and atherogenic profiles [74–76]. A 2025 review emphasized the plausibility of cardiovascular risk factors as causal drivers of both AD (including congophilic angiopathy) and vascular dementia, supported by converging epidemiological, imaging, and neuropathological evidence [77]. Similarly, vascular burden is considered an early contributor to AD pathogenesis, reinforcing the concept that vascular health in midlife may play a decisive role in cognitive outcomes later in life [78].

Longitudinal and population-based studies have further explored this association. In this regard, an early work aimed at evaluating whether sleep-disordered breathing predicted cognitive decline in older women showed that individuals with sleep-disordered breathing exhibited a higher likelihood of developing cognitive impairment compared with those without the condition [79]. In addition, evidence from a male veteran cohort indicated that sleep disturbances were significantly associated with an increased incidence of dementia [80]. An association between OSA and dementia risk has also been demonstrated in a nationwide 5-year population-based study in Taiwan [81]. Population evidence further illustrates a bidirectional relationship. In this context, a meta-analysis by Emamian et al. reported that patients with AD have nearly a fivefold higher risk of presenting with OSA compared with age-matched controls, and approximately 50% of AD patients develop OSA following diagnosis [82]. Another meta-analysis concluded that sleep-disordered breathing is associated with cognitive impairment [83]. A systematic review and meta-analysis including more than 1.3 million participants found that OSA was associated with an increased risk of AD (HR 1.28, 95% CI 1.16–1.41) and of any neurocognitive disorder (HR 1.43, 95% CI 1.26–1.62) [84]. Findings from a Danish population-based investigation corroborates a modest link between OSA and dementia, including AD, and emphasize the public health relevance of early detection and treatment of OSA as a modifiable contributor to risk [85]. In this regard, a very recent study using data from 2.3 million adults concluded that OSA is associated with higher risks of all-cause dementia and several of its subtypes [86].

Interestingly, some evidence points to sex-related differences in the association between OSA and dementia. Women are disproportionately affected by AD, partly due to longer life expectancy. However, research suggests that additional biological and sociocultural factors contribute to this disparity, including neurohormonal changes at menopause, genetic influences, and differences in education or life experience [2]. Preclinical evidence from Cheung et al. demonstrated a strong association between intermittent hypoxia and cognitive decline in ovariectomized female rats, mediated by increased hippocampal tau levels [87]. These findings align with a longitudinal cohort study of 18,815 older adults showing that the cumulative incidence of dementia by age 80 was 4.7% higher in women with confirmed or suspected OSA, compared with a 2.5% increase in men, suggesting heightened vulnerability in aging women [88].

However, there is also complementary evidence, and not all studies are aligned with these interpretations. In a population cohort of 2,636 community-dwelling older men (mean age 76.0 ± 5.3 years), free of probable mild cognitive impairment or dementia and followed for an average of 3.4 ± 0.5 years, no significant association between apnea–hypopnea index and cognitive decline was found [89]. Furthermore, no significant longitudinal changes in executive or memory functions were observed in a cohort of healthy elderly subjects [90]. Lutsey et al. [91], in a cohort study of approximately 1,700 individuals followed for more than 15 years, reported that OSA showed no association with dementia when using broad diagnostic codes. However, in participants who underwent detailed neurocognitive assessments, severe OSA was linked to higher risk of all-cause and AD dementia, although this association was attenuated after adjustment for lifestyle and cardiovascular factors, suggesting mediation through vascular pathways.

As discussed above, the pathophysiological features of OSA create a systemic environment of oxidative stress and inflammation. One of the key mediators linking OSA to downstream cardiovascular, metabolic, and neurological consequences is the overproduction of reactive oxygen species (ROS). In parallel, oxidative stress has long been recognized as a central contributor to AD pathogenesis, suggesting that ROS may represent a mechanistic bridge between OSA and neurodegeneration [55, 92]. Beyond vascular consequences, ROS directly impair neuronal homeostasis by damaging mitochondrial DNA, altering synaptic proteins, and compromising calcium handling. These processes are particularly deleterious in the aging brain, where endogenous antioxidant defenses are diminished [92].

In the context of AD, oxidative stress is both an upstream trigger and a downstream amplifier of neuropathology. ROS promote the aggregation and impaired clearance of Aβ, while also enhancing hyperphosphorylation of tau, thereby accelerating the formation of neurofibrillary tangles [55, 93]. Conversely, Aβ itself can stimulate microglia and astrocytes to release additional ROS, perpetuating a vicious cycle of oxidative injury [53]. Moreover, oxidative stress impairs the glymphatic clearance system, further reducing elimination of Aβ and tau from the brain [70]. Thus, ROS may occupy a central position in the self-propagating loop of AD pathology. All the above epidemiological and clinical studies provide indirect support for a mechanistic link. Patients with OSA exhibit higher circulating and cerebral markers of oxidative stress, including elevated malondialdehyde, 8-hydroxy-2′-deoxyguanosine, 8-isoprostane, and oxidized lipoproteins [74, 94–96]. These markers correlate with both disease severity and neurocognitive impairment. Importantly, increased oxidative stress is also observed in CSF and plasma from individuals with early AD or mild cognitive impairment, supporting the notion of a shared pathophysiological substrate [97]. Experimental models reinforce this connection: mice exposed to chronic intermittent hypoxia display memory deficits, elevated Aβ₄₂ accumulation, and enhanced tau phosphorylation, all of which are accompanied by heightened oxidative damage in hippocampal and cortical regions [18, 98, 99].

The therapeutic implications of an association of AD and OSA are significant, and relevant to both patients and their careers. Continuous positive airway pressure (CPAP), the standard treatment for OSA, has been shown not only to improve oxygenation and sleep quality but also to partially reduce oxidative stress markers in blood and neural tissue [74, 100, 101]. CPAP in OSA could present both immediate and long-term neurological benefits. In the short term, CPAP enhances sleep efficiency and improves daytime alertness and may support better executive functioning. Over the long term, CPAP use is independently associated with a reduced risk of AD in older adults, suggesting a potential influence on the underlying neurodegenerative mechanisms [102]. Furthermore, recent findings indicate that it leads to measurable cognitive improvements and a global increase in cerebral glucose metabolism, as observed via 18F-FDG PET imaging [103]. Despite these promising outcomes, it remains unclear whether CPAP directly enhances neuronal integrity or slows the progression of neurodegeneration. Antioxidant strategies, including supplementation with vitamins C and E, polyphenols, and agents targeting mitochondrial redox balance, have been explored in both OSA and AD, though clinical results remain inconsistent [104–108]. In summary, the convergence of evidence indicates that ROS generated during OSA may contribute to the initiation and progression of AD pathology. By linking intermittent hypoxia to oxidative neuronal damage and amyloid–tau dynamics, ROS provide a plausible mechanistic explanation for the mechanistic association between OSA and AD. Targeting oxidative stress, through both OSA management and adjunctive antioxidant approaches, represents a promising avenue for reducing the neurodegenerative burden associated with OSA in aging populations.

Experimental data provide support for this association. In triple-transgenic AD mouse models, chronic intermittent hypoxia induced increased levels of Aβ42 but not Aβ40 in the brains of mice, although no significant changes in cognitive function were observed [109]. Evidence from CSF biomarkers of AD further reinforces this view. A longitudinal study with two years of follow-up showed that baseline severity of OSA predicted a more rapid reduction in CSF Aβ42. In contrast, another study demonstrated that exposure to chronic hypoxia reduced cognitive and memory function in AD mouse models and was associated with both increased senile plaque deposition and enhanced tau phosphorylation [110]. On the contrary, studies in young wild-type animals did not show any impact on the transcription of Aβ-related genes nor significant modulation of Bace-1, AβPP, or the C99/C83 ratio. Furthermore, chronic sustained hypoxia did not significantly alter the expression of Aβ40, Aβ42, AβPP, or sAβPPα in either young or aged APP/PS1 mice [111].

Taken together, a growing body of basic, clinical, epidemiological, and mechanistic research converges on a central idea: astrocytic dysfunction, oxidative stress, and sleep-related hypoxia are closely interconnected contributors to Alzheimer’s disease. Intermittent hypoxia, traditionally viewed as a secondary consequence of sleep apnea, is now recognized as a factor capable of altering astrocytic oxygen sensing and redox signaling, with direct effects on metabolic balance, vascular regulation, and inflammatory responses. These observations place astrocytes at the center of a biological axis linking a highly prevalent systemic disorder with neurodegeneration. More importantly, they suggest that OSA is not simply a coexisting condition but a modifiable risk factor that may accelerate Alzheimer-related pathology.

Thus, in a field where effective disease-modifying treatments remain limited, this connection highlights a valuable opportunity: reducing hypoxia-induced astrocytic dysfunction may help preserve cognitive function and slow neurodegenerative progression in aging individuals. Recognizing this link and integrating it into clinical practice could significantly strengthen dementia-prevention strategies in the coming decades.

## Data Availability

This manuscript has not associated data.
